# Features Analysis of Lower Extremity Arterial Lesions in 162 Diabetes Patients

**DOI:** 10.1155/2013/781360

**Published:** 2013-04-15

**Authors:** XiangJiang Guo, YaXue Shi, XiaoZhong Huang, Meng Ye, GuanHua Xue, JiWei Zhang

**Affiliations:** Department of Vascular Surgery, Renji Hospital, School of Medicine, Shanghai Jiaotong University, Shanghai 200001, China

## Abstract

*Objective*. This study aimed to investigate the angiographic manifestations of lower extremity atherosclerotic steno-occlusive disease in patients with diabetes. *Materials and Methods*. A total of 162 patients with diabetes were enrolled in this study. The angiographic findings of lower extremity arterial lesions were evaluated according to location (iliac, femoral, popliteal, and crural artery), type (stenosis or occlusion), and length (<5 cm, 5–10 cm, and >5 cm). *Results*. A total of 131 of 162 (80.9%) diabetics showed multiple segmental lesions, and 19.1% (31/162) presented single segmental lesions in the lower extremity artery. Crural artery was the mainly involved location (39/162, 85.8%). Among the recorded 660 lesions of 162 cases, 437 (66.2%) were occlusion lesions, while 223 (33.8%) were stenosis lesions. Of 437 occlusion lesions, 308 lesions (70.5%) were in crural artery. More than 10 cm occlusion lesion (242/392, 61.7%) was the main manifestation in crural artery, especially in anterior (92/127, 67.2%) and posterior tibial arteries (91/124, 73.4%), which was higher than that in iliac artery (8/33, 24.2%), popliteal artery (53/157, 33.8%), and femoral artery (11/78, 14.1%). *Conclusion*. In diabetic subjects with lower limb artery ischemia, the vascular involvement is extremely diffuse and particularly severe in crural arteries, with high prevalence of more than 10 cm occlusion lesions.

## 1. Introduction

 The complications of diabetic vasculopathy commonly include two categories: microvascular and macrovascular complications. Macrovascular disease is the most common reason of mortality and morbidity in diabetes and is responsible for high incidence of vascular diseases such as stroke, myocardial infarction, and peripheral vascular diseases (PAD) [1]. Epidemiological evidence has confirmed an association between diabetes and increased prevalence of PAD. The duration and severity of diabetes correlate with incidence and extent of PAD [2, 3]. Diabetes changes the nature of PAD. Traditionally, macrovascular diseases are thought as underlying obstructive atherosclerotic diseases of major arteries. Diabetic patients more commonly have infrapopliteal arterial occlusive disease and vascular calcification than nondiabetic cohorts [4]. Pathological changes of major blood vessels lead to functional and structural abnormalities in diabetic vessels including endothelial dysfunction, reduced vascular compliance, and atherosclerosis [5]. Diabetic lower extremity arterial disease is the main cause of nontraumatic amputation. Diabetic patients with lower extremity vascular disease have a higher prevalence, 20 times higher than that of the nondiabetic patients. Approximately, 8% of diabetic patients have already presented lower extremity arterial disease before a definite diagnosis. So, it is important to study diabetic patients with peripheral artery disease. This paper aimed to explore the involved lower extremity arterial lesions in diabetic patients and its characteristics.

## 2. Materials and Methods

### 2.1. Objects

The experiments were conducted with the understanding and written consent of each subject and were approved by the responsible Ethical Committee. One hundred and sixty-two diabetes patients with lower limb artery ischemia were admitted to our hospital from January 2007 to December 2008. Inclusion criteria were (1) lower limb artery ischemia patients with more than grade 2 in the Fontaine classification; (2) patients diagnosed as diabetics and undergoing parallel oral hypoglycemic drugs or insulin replacement therapy; (3) nonthrombotic and nonembolic lesions; (4) patients having no history of interventional or surgical vascular bypass surgery.

Among 162 diabetic patients with lower limb artery ischemia that met the inclusion criteria, there were 83 males and 79 females, aged from 49 to 93 years old with the average of 76. In 90 cases, the lesion occurred on the left limb, while in 72 cases the lesion occurred on the right limb. The diagnosis course of diabetes varied from 3 months to 28 years with an average of 7.6 years. According to Fontaine classification, there were 44 cases of grade II, 47 cases of grade III, and 71 cases of grade IV. The major risk factors included abnormal lipid metabolism, hypertension, coronary heart disease, cerebral infarction, uremia, and smoking (see Table 1).

### 2.2. Methods

The affected limbs of enrolled patients were examined with digital subtraction angiography (DSA) routinely by contralateral retrograde puncture in the following order: iliac artery, femoral artery, popliteal artery, and crural artery. The location of the lesion may be iliac (common iliac artery and external iliac artery), femoral (common femoral artery, superficial femoral artery, and profunda femoris), popliteal, and crural (anterior tibial, posterior tibial, and peroneal) as described by Graziani et al. [6]. According to the results of DSA, the lesion may be single-segment or multilevel arterial occlusive disease (MLAOD) which is defined as the lesions in adjacent segments such as the iliac and femoral artery lesions or ≥2 segments lesions of ipsilateral lower extremity arteries such as femoral-crural artery lesions or iliac-femoral-popliteal artery lesions [7]. The length of each encountered lesion was categorized into three groups: <5 cm, 5–10 cm, and >10 cm. Stenosis was defined by a reduction of lumen diameter between 50 and 99%. Occlusion was defined by a total obliteration of the lumen. Each lesion was then statistically categorized according to location, type (stenosis or occlusion), and length. 

### 2.3. Statistical Analysis

All data were calculated by SAS8.02 statistical analysis software. Measurement data were expressed as mean ± standard deviation and analyzed by *t*-test, while count data were analyzed using Fisher's exact test and chi-square (*χ*2) test. *P* < 0.05 was considered statistically significant.

## 3. Results

As shown in Table 2, in 162 cases with affected limbs, 31 patients (19.1%) had single segmental lesions, 70 patients (43.2%) had the two segmental lesions, and 61 cases (37.7%) had three or more segmental lesions. Among 31 cases of single segmental lesions, there were 19 cases of crural artery lesions, 8 cases of femoral artery lesions, and 4 cases of iliac artery lesions. Among 70 cases of two segmental lesions, 43 cases of femoral-crural artery lesions, 19 cases of popliteal-crural artery lesions, 6 cases of iliac-femoral artery lesions, and 2 cases of femoral-popliteal artery lesions. Among 61 cases of three or more segmental lesions, there were 41 cases of femoral-popliteal-crural artery lesions, 12 cases of iliac-femoral-crural artery lesions, 3 cases of iliac-femoral-popliteal artery lesions, and 5 cases of four segmental lesions. Furthermore, we found that lesions of 139 cases (85.8%) were in crural artery, 120 cases (75.0%) were in femoral artery, 30 cases (18.5%) were in iliac artery, and 70 cases (43.2%) in the popliteal artery. Obviously, the occurrence rate of lesions in crural artery was higher than that in femoral artery, iliac artery, and popliteal artery. 

Among the recorded 660 lesions of 162 cases, 437 (66.2%) were occlusion lesions, while 223 (33.8%) were stenosis lesions. Of 437 occlusion lesions, 308 lesions (70.5%) were in crural artery. There were, respectively 8, 11, 53, and 242 occlusions >10 cm in iliac artery, popliteal artery, femoral artery, and crural artery. This indicated that occlusion >10 cm was mainly distributed in crural arteries followed by femoral artery. The difference between arterial segments was statistically significant (*P* < 0.01) (Table 3). In addition, the difference of occlusion >10 cm among three sections of crural artery (anterior tibial, posterior tibial, and peroneal artery) was also statistically significant (*P* < 0.01) (Table 4).

In 157 lesions involved in the femoral artery, only 3 common femoral artery lesions and 2 deep femoral artery lesions were observed. In lesions of superficial femoral artery, superficial femoral artery ostia (49/157, 31.2%) and adductor tube (56/157, 35.7%) had been mainly involved.

## 4. Discussion and Conclusion

Our findings show that in diabetic subjects with lower limb artery ischemia, the vascular involvement is extremely diffuse and particularly severe in crural arteries (139 cases, 85.8%). More than 10 cm occlusion lesion (242/392, 61.7%) was the main manifestation in crural artery, of which anterior tibial (92/137, 67.2%) and posterior tibial arteries (91/124, 73.4%) are clinically more relevant. These are consistent with the previous study [6].

Diabetes is closely related with atherosclerosis, showing that the diabetics present earlier and progressive worsening atherosclerosis symptoms, as well as poor prognosis. The interaction between metabolic and hemodynamic factors is the pathogenesis of diabetic vascular complications. Recent studies have found that type 2 diabetes and atherosclerosis may be parallelly developed from the same pathological changes [8], the common basis of which is the subacute, non-infectious inflammatory response caused by excessive oxidative stress. If the previous process occurs in pancreatic *β*-cells, it can cause abnormal insulin secretion, but, leads to insulin resistance when in muscle and adipose tissue and can cause the activation of the atherosclerosis when in vascular endothelial cells. High blood sugar, insulin resistance, dyslipidemia, and other factors lead to the vascular endothelial cell injury, a decrease in nitric oxide synthesis, bioavailability, and an increase in degradation, ultimately resulting in inhibition of nitric oxide-mediated vasodilation function [9]. Diabetic lower extremity arterial medial calcification (Mönckeberg calcification) aggravates vascular sclerosis. Usually, luminal diameter is maintained through compensatory increase of vascular circumference in the process of atherosclerosis (Glagov phenomenon). All the previous pathological changes alter artery remodeling process, leading to diffuse stenosis of the end of vessels and the decline of compensatory vascular expansion function [10]. So, distal artery of the limbs (such as crural arteries) is the main involved artery in diabetics with lower extremity atherosclerosis in contrast to the proximal arterial lesions such as the iliac and femoral artery atherosclerosis in nondiabetics with lower extremity atherosclerosis, the latter can be caused mainly by hypertension and hemodynamic factors.

The treatment of long segment arterial occlusion is challenging and thus is the focus of study of lower extremity arterial occlusive disease [11]. Peroneal artery is not the direct feeding artery of the foot, but it can constitute a rich collateral circulation and become the most important compensatory blood supply artery by the extensive linking with the medial malleolus network, lateral malleolus network, calcaneal rete, as well as anterior tibial and posterior tibial arteries in the ankle area. Therefore, in the treatment of patients with critical limb ischemia (CLI), whatever by surgical bypass surgery or lumen interventional treatment, peroneal artery can be considered as the beats outflow tract. Dosluoglu et al. [12] report excellent effect in the endovascular treatment of ischemic lower extremity ulcer and gangrene when the peroneal artery is regarded as the only outflow tract. There is no significant difference in flow patency rate, limb salvage rate, and ulcer healing time when compared with anterior tibial and posterior tibial arteries as the outflow tract. Our results also showed lower long segment arterial occlusion rate in the peroneal artery.

 Diabetics with lower extremity arterial disease mainly present with MLAOD, but only with small ratio of single segmental lesions. In this study, 131 of 162 limbs (80.9%) had more than two segmental lesions compared with 19.1% (31/162) single segmental lesions. In MLAOD, femoral artery and crural artery were the most common lesions locations, followed by popliteal artery and iliac artery. Of two segmental lesions, the femoral-crural artery (43/70, 61.4%) was mainly involved, while 67.2% (41/61) femoral-popliteal-crural artery lesions were observed in the three segmental lesions. According to the principle of Poiseuille [13], the changes in the radius of the blood vessel need more fluid energy than those in the length of the blood vessel. Therefore, the multilevel arteriosclerosis inevitably leads to a huge loss of blood energy. Additionally, due to vascular sclerosis, microthrombi are easily formed, and severe ischemia of the limbs was ultimately developed.

The study found that the femoral artery (120/162, 75.0%) was the second involved location in diabetics with lower extremity arterial lesions, of which the superficial femoral artery was more mainly involved rather than common femoral artery and deep femoral artery. This was not consistent with the previous studies that report deep femoral artery being mostly involved [14–16]. Generally, atherosclerotic occlusive lesions occur in artery bifurcation, and bending or pressure parts of main artery such as superficial femoral artery ostia and the adductor tube because of the following hemodynamic factors: the vortex formation under mechanical force by the impact of blood flow to the arterial intima; intimal injury, platelet deposition and thrombosis when blood vessel wall under sustained shear pressure [17]; the convex inner wall of these artery bifurcations and bending likely forms a relatively negative pressure partially due to the direction changes of blood flow, thus promoting plaque formation easily. In addition, the unique anatomy of the superficial femoral artery adductor tube is an important reason leading to arterial occlusion [18]. Adductor tube is a potential tubular structure in the lower part of the thigh surrounded by vital muscle group dominating the lower extremity activity, inside which femoral vascular sheath passes. At the tendinous opening of the adductor magnus, the medial wall of femoral vascular sheath is adjacent to toughed adductor magnus tendon, and the lateral wall of femoral vascular sheath is adjacent to the medial femoral condyle, the two structures of which are so hard as to cause mechanical compression of the adductor tube. Repeated contraction of the surrounding muscles with the movement of the lower limbs enables arterial wall thickening and lumen narrowing gradually and leads to arterial occlusion with subsequent thrombosis. The tendinous opening of the adductor magnus is the predilection site of stenosis and occlusion.

## Figures and Tables

**Table 1:** General condition of 162 diabetic patients.

Parameter	Cases (%)
Hypertension	95 (58.6)
Coronary heart disease	33 (20.4)
Cerebral infarction	52 (32.1)
Abnormal lipid metabolism	107 (66.1)
Uremia	7 (4.3)
Smoking	45 (27.8)
Fontaine classification	
II	44 (27.2)
III	47 (29.0)
IV	71 (43.8)

**Table 2:** Location of the lower limb artery lesion in 162 diabetic patients.

Lesions (*n*, %)	Location	Cases (*n*, %)
Total (*n* = 162)	Crural artery	139/162, 85.8%
	Femoral artery	120/162, 75.0%
	Iliac artery	30/162, 18.5%
	Popliteal	70/162, 43.2%

Single segmental (31/162, 19.1%)	Crural artery	19/31, 61.3%
	Femoral artery	8/31, 25.8%
	Iliac artery	4/31, 12.9%

Two segmental (70/162, 43.2%)	Femoral-crural	43/70, 61.4%
	Popliteal-crural	19/70, 27.1%
	Iliac-femoral	6/70, 8.6%
	Femoral-popliteal	2/70, 2.9%

≥3 segmental (61/162, 37.7%)	Femoral-popliteal-crural artery	41/61, 67.2%
	Iliac-femoral-crural artery	12/61, 19.7%
	Iliac-femoral-popliteal artery	3/61, 4.9%
	Iliac-femoral-popliteal-crural artery	5/61, 8.2%

**Table 3:** Length and type of the lower limb artery lesion in 162 diabetic patients.

Location	<5 cm		5–10 cm		>10 cm		Total cases	Rate of >10 cm occlusion segments (%)
	Stenosis	Occlusion	Stenosis	Occlusion	Stenosis	Occlusion		
Iliac artery	12	2	6	4	1	8	33	24.2
Femoral artery	46	12	17	17	12	53	157	33.8
Popliteal artery	31	9	9	13	5	11	78	14.1
Crural artery	35	21	26	45	23	242	392	61.7

There was statistically significant occlusion >10 cm between the four arteries (*P* < 0.01).

**Table 4:** The involvement extent of crural artery lesions.

Location	<5 cm		5–10 cm		>10 cm		Total cases	Rate of >10 cm occlusion segments (%)
	Stenosis	Occlusion	Stenosis	Occlusion	Stenosis	Occlusion		
Anterior tibial artery	11	8	7	10	9	92	137	67.2
Posterior tibial artery	7	6	7	9	4	91	124	73.4
Peroneal artery	17	7	12	26	10	59	131	45.0

The difference of occlusion >10 cm among these three sections was statistically significant (*P* < 0.01), but *P* = 0.2719 between anterior and posterior tibial arteries, *P* < 0.01 between anterior tibial artery and peroneal artery, and *P* < 0.01 between posterior tibial artery and peroneal artery.
